# PRO: Do We Still Need Whole-Brain Irradiation for Brain Metastases?

**DOI:** 10.3390/cancers15123193

**Published:** 2023-06-15

**Authors:** Ilinca Popp, Nanna E. Hartong, Carsten Nieder, Anca-L. Grosu

**Affiliations:** 1 Department of Radiation Oncology, Medical Center—University of Freiburg, Faculty of Medicine, University of Freiburg, 79106 Freiburg, Germany; 2 German Cancer Consortium (DKTK), German Cancer Research Center (DKFZ), Partner Site Freiburg, 69120 Heidelberg, Germany; 3 Department of Oncology and Palliative Medicine, Nordland Hospital, 8092 Bodø, Norway; 4 Department of Clinical Medicine, Faculty of Health Sciences, UiT—The Arctic University of Norway, 9037 Tromsø, Norway

**Keywords:** WBRT, brain metastases, hippocampus avoidance, cognition

## Abstract

**Simple Summary:**

Whole-brain radiation therapy (WBRT) has been a mainstay in the treatment of multiple cerebral metastases for many decades. However, evidence of its negative effects on cognitive functions and quality of life has rendered conventional WBRT unwanted and led to widespread use of local stereotactic therapies instead. However, newer WBRT methods have been proven to be efficient and safe and have become readily available in past years. No clinical trials have compared the oncological and functional outcomes of multiple radiosurgeries with these newer methods. The available data suggest that modern WBRT techniques can play a significant role in the treatment of multiple brain metastases and warrant further prospective research.

**Abstract:**

(1) Background: In recent decades, the use of whole-brain radiation therapy (WBRT) in the treatment of brain metastases has significantly decreased, with clinicians fearing adverse neurocognitive events and data showing limited efficacy regarding local tumor control and overall survival. The present study thus aimed to reassess the role that WBRT holds in the treatment of brain metastases. (2) Methods: This review summarizes the available evidence from 1990 until today supporting the use of WBRT, as well as new developments in WBRT and their clinical implications. (3) Results: While one to four brain metastases should be exclusively treated with radiosurgery, WBRT does remain an option for patients with multiple metastases. In particular, hippocampus-avoidance WBRT, WBRT with dose escalation to the metastases, and their combination have shown promising results and offer valid alternatives to local stereotactic radiotherapy. Ongoing and published prospective trials on the efficacy and toxicity of these new methods are presented. (4) Conclusions: Unlike conventional WBRT, which has limited indications, modern WBRT techniques continue to have a significant role to play in the treatment of multiple brain metastases. In which situations radiosurgery or WBRT should be the first option should be investigated in further studies. Until then, the therapeutic decision must be made individually depending on the oncological context.

## 1. Introduction

Brain metastases are the most frequent type of intracranial tumors and occur in up to a third of systemic malignancies [1]. With an initial median survival of 3–6 months [2], the prognosis of brain metastases has always been the most important endpoint in interventional clinical trials. However, with median survival rates increasing to 13 months at present [3], post-therapeutic changes in neurocognitive functions and quality of life have now gained more and more importance.

Whole-brain radiation therapy (WBRT) has been a mainstay in the treatment of brain metastases for over 60 years. However, evidence of significant neurocognitive decline following WBRT has led to a general tendency to replace WBRT with local therapies, such as stereotactic radiosurgery (SRS), even in the case of extensive brain metastatic disease. In accordance with this trend, the most recent ASCO-SNO-ASTRO recommendations placed multiple SRSs as the first option for all fit patients with up to 10 brain metastases, with WBRT only being endorsed for patients with poor performance status or with metastases that are not suitable for SRS [4].

As a result of these developments in past years, WBRT has definitely started to be considered an “old-fashioned” treatment option. Nevertheless, it does remain an important alternative, even in the current guidelines. We consider that there is a place and time for using WBRT, and we therefore aimed to investigate the available literature in order to identify the role WBRT still holds in the therapy of brain metastases.

## 2. Whole-Brain Radiation Therapy for Limited Brain Metastatic Disease

Initially, all patients with brain metastases were treated with WBRT as the standard of care. However, for patients with a limited number of brain metastases (one to four), several randomized trials made it apparent that local therapies—surgical resection and stereotactic radiotherapy—are a more appropriate alternative in almost all cases (Table 1).

Especially for larger lesions causing mass effects and neurological symptoms, surgical resection plus radiotherapy has been associated with longer overall and progression-free survival, as well as better quality of life, than WBRT alone [5,6]. SRS and stereotactic fractionated radiotherapy (SFRT) to the surgical bed led to local tumor control rates of 70–90% at one year, the same overall survival, and an improved safety profile [7,8,9,10] and thus replaced WBRT as the standard postoperative treatment [11].

For metastases amenable to primary radiotherapeutic treatment, SRS also resulted in less cognitive deterioration without negatively impacting overall survival rates as compared to WBRT [12,13,14].

All in all, WBRT does not bring a survival benefit for patients with one to four brain metastases (hazard ratio = 1) [15] and should therefore be omitted for patients with good performance status. However, it is noticeable from the available prospective data (Table 1) that WBRT is superior to SRS in reducing distant intracranial tumor progression (hazard ratio = 2.34 [15]) and neurological death rates [14,16,17]. Moreover, WBRT may still have a role to play in the treatment of patients with extensive extracranial disease [4], as delaying neurological symptoms and the need for salvage therapies can outweigh the risks of neurocognitive decline because of limited life expectancy.

Furthermore, it should be noted that, in these studies, addition of WBRT to SRS led to significantly better local tumor control rates than SRS alone (hazard ratio = 2.73 [15]). Whether this was a consequence of the total cumulative dose, poor targeting, or metastatic infiltration at the interface between the metastasis and brain parenchyma is unclear and should be further explored.

**Table 1:** Available evidence regarding whole-brain radiation therapy for one to four brain metastases.

Trial	Design	Patients	Oncological Outcome
Patchell et al., 1998 [16]	OP + WBRT vs. OP + Obs	49 vs. 46	Local recurrence: 10% vs. 46%, *p* < 0.001 *
			Distant recurrence: 14% vs. 37%, *p* < 0.01 *
			Median OS: 48 vs. 43 weeks, *p* = 0.39
			Neurological death: 14% vs. 44%, *p* = 0.003 *
Aoyama et al., 2006 [12]	WBRT + SRS vs. SRS	65 vs. 67	One-year recurrence: 46.8% vs. 76.4%, *p* < 0.001 *
			Median OS: 7.5 vs. 8.0 months, *p* = 0.42
			Neurological death: 22.8% vs. 19.3%, *p* = 0.64
Kocher et al., 2011 [17]	(SRS vs. OP) + WBRT vs. (SRS vs. OP) + Obs	(99 vs. 81) vs. (100 vs. 79)	Two-year local recurrence (OP): 59% vs. 27%, *p* < 0.001 *
			Two -year local recurrence (SRS): 31% vs. 19%, *p* = 0.040 *
			Two -year distant recurrence (OP): 42% vs. 23%, *p* = 0.008 *
			Two -year distant recurrence (SRS): 48% vs. 33%, *p* = 0.023 *
			Median OS: 10.9 v 10.7 months, *p* = 0.89
			Neurological death: 44% vs. 28%, *p* < 0.002 *
El Gantery et al., 2014 [13]	SRS vs. WBRT vs. SRS + WBRT	18 vs. 21 vs. 21	Median LTC: 6 vs. 5 vs. 10 months, *p* = 0.04
			Median OS: no significant difference
			Median OS (BMs < 3 cm): 8 vs. 5 vs. 15 months, *p* = 0.002
Kayama et al., 2018 [18]	OP + Obs + salvage SRS vs. WBRT	134 vs. 137	Median PFS: 4.0 vs. 10.4 months *
			Median OS: 15.6 vs. 15.6 months, *p* = 0.027
			Neurological death: 21.0% vs. 21.9%
Brown et al., 2016 [14]	SRS vs. SRS + WBRT	111 vs. 102	Six-month LTC: 81.6% vs. 92.6%, *p* = 0.034 *
			Twelve-month LTC: 72.8% vs. 90.1%, *p* = 0.003 *
			Six-month DTC: 76.7% vs. 94.7%, *p* < 0.001 *
			Twelve-month DTC: 69.9% vs. 92.3%, *p* < 0.001 *
			Median OS: 7.4 vs. 10.4 months, *p* = 0.92
Brown et al., 2017 [8]	OP + SRS vs. OP + WBRT	98 vs. 96	Six-month LTC: 80.4% vs. 87.1%, *p* = 0.00068 *
			Median PFS: 6.4 vs. 27.5 months, *p* < 0.0001 *
			Median OS: 12.2 vs. 11.6 months
Palmer et al., 2022 [19]	OP + SRS vs. OP + WBRT	27 vs. 27	Twelve-month LTC + DTC: 81.5% vs. 40.7% *

OP—operative resection, WBRT—whole-brain radiation therapy, SRS—stereotactic radiosurgery, Obs—observation, LTC—local tumor control, DTC—distant intracranial control, OS—overall survival, PFS—progression-free survival, BM—brain metastasis, SD—standard deviation. * Oncological outcomes in favor of WBRT.

## 3. Whole-Brain Radiation Therapy for Extensive Brain Metastatic Disease

Patients with a higher number of brain metastases have a greater brain metastasis velocity, signifying a higher risk of developing new metastases and a worse prognosis [20]. Therefore, the advantage of improved distant tumor control through WBRT would be put to best use in cases of multiple (more than four) metastases. However, WBRT is unquestionably associated with significant neurocognitive deficits, as well as further adverse events, such as gait instability and fatigue [12,14,19,21,22]. Cognitive deterioration following WBRT has been found to be clinically meaningful among long-term survivors and to significantly impact quality of life [19]. This has led to reluctance toward using conventional WBRT for any purpose, especially in patients with high performance status, good prognoses, and available targeted therapies crossing the blood–brain barrier [4].

In support of this reluctance came the prospective randomized QUARTZ trial, which investigated the best supportive care plus WBRT versus the best supportive care alone in cerebrally metastasized non-small cell lung cancer patients [23]. The trial showed no differences in overall survival, quality of life, or dexamethasone use between the two groups. However, the radiation dose used in this trial was 20 Gy in five daily fractions, which equates to a biologically effective dose of 28 Gy to the tumor and is the equivalent of 23 Gy in 2 Gy fractions with an alpha/beta of 10. Since there appears to be a dose–effect relationship in the treatment of brain metastases [24], it is possible that the applied dose was insufficient. Furthermore, patients were only included if they did not qualify for stereotactic treatment or surgical resection, with two thirds having one to four brain metastases, almost 80% being over the age of 60, and over one third of them having a poor Karnofsky performance status of 30–70%. The results are therefore not surprising taking into consideration the low dose for the WBRT and the negative selection of the patient cohort. However, when looking only at those patients younger than 60 years, those with better prognostic scores, and those with five or more brain metastases, the trial did find a tendency for improved quality of life-adjusted survival with WBRT as compared to best supportive care alone.

### 3.1. Hippocampal Irradiation and Cognitive Impairment

Neurocognitive deficits—more precisely, episodic and verbal memory dysfunctions—have been observed early in the follow-up after WBRT [25]. This has been mainly attributed to the irradiation of the hippocampus, the central structure responsible for learning and memory storage. The two hippocampi have remarkable plasticity achieved through long-term potentiation [26] and include stem-cell niches responsible for ongoing adult neurogenesis [27]. The hippocampi are functionally, anatomically, and cytoarchitecturally different from the cerebral cortex [28] and much more prone to radiation-induced atrophy in comparison to other structures [29]. Low doses of radiation to the hippocampi have been shown to be enough to induce persistent neuroinflammation, with subsequent inhibition of neurogenesis and atrophy [30,31]. While multiple other cerebral structures are also involved in higher-order cognitive functions, they are less susceptible to low and moderate doses of radiation in comparison to the hippocampus [29].

Rates of hippocampal atrophy have been used as both diagnostic and prognostic markers in clinical trials of Alzheimer’s disease and mild cognitive impairment, with higher rates correlating with higher degrees of memory dysfunction [32,33]. The relevance of radiotherapy-induced hippocampal atrophy for cognitive functions has only been studied in a few publications, but their results did reflect the data from the neurodegenerative disease research [34,35,36]. Since there appears to be a correlation between dose, atrophy, and cognition, approaches to protect the hippocampi have been developed and implemented in clinical trials. These include the concomitant administration of neurocognitive protecting agents, hippocampus-avoidance WBRT (HA-WBRT), and HA-WBRT with a reduced whole-brain dose and simultaneous integrated boost (SIB) to the metastases (HA-WBRT + SIB).

### 3.2. Whole-Brain Radiation Therapy and Concomitant Memantine Administration

The administration of neurocognitive protective agents during WBRT relies on the hypothesis that radiation-induced toxicity is similar in pathophysiology to the small vessel disease seen in vascular dementia. Vascular injury leads to local ischemia, inducing cellular damage through excessive N-methyl-D-aspartate (NMDA) stimulation [37]. Memantine is an uncompetitive antagonist of glutamate NMDA receptors mostly used for Alzheimer’s disease and mild-to-moderate vascular dementia [38], and it has been hypothesized to help reduce side effects of WBRT [39].

There are various clinical trials that have examined the efficacy of memantine in preventing neurocognitive side effects in patients being treated with WBRT. Brown et al. randomized 554 patients from 143 centers in the United States and Canada to memantine versus placebo. The use of memantine resulted in better cognitive function over time, but no statistically significant difference was seen [39]. Laack et al. similarly randomized 508 participants to memantine versus placebo and also found no significant difference between arms [40]. However, promising preclinical data have shown that memantine can prevent radiation-induced synaptic remodeling [41], and imaging studies with dynamic contrast-enhanced MRI found reduced WBRT-induced cerebral vascular damage after memantine administration [42].

Since randomized trials have not achieved statistical significance, opinions on the efficacy of memantine remain controversial. Memantine is available as 5 mg and 10 mg oral tablets. The prescribed dose for patients with brain metastases receiving WBRT starts with 5 mg/day in the first days of radiotherapy and increases in weekly 5 mg increments over 4 weeks to the final dose of 20 mg/day, which is maintained over the course of 20 weeks [39]. Its use appears to be more frequent in USA-based medical facilities than in Europe.

### 3.3. Hippocampus-Avoidance Whole-Brain Radiation Therapy

A more promising technique that has gained worldwide recognition is the sparing of the hippocampus in WBRT planning. The clinical trials investigating this method are presented in Table 2.

**Figure 1:**
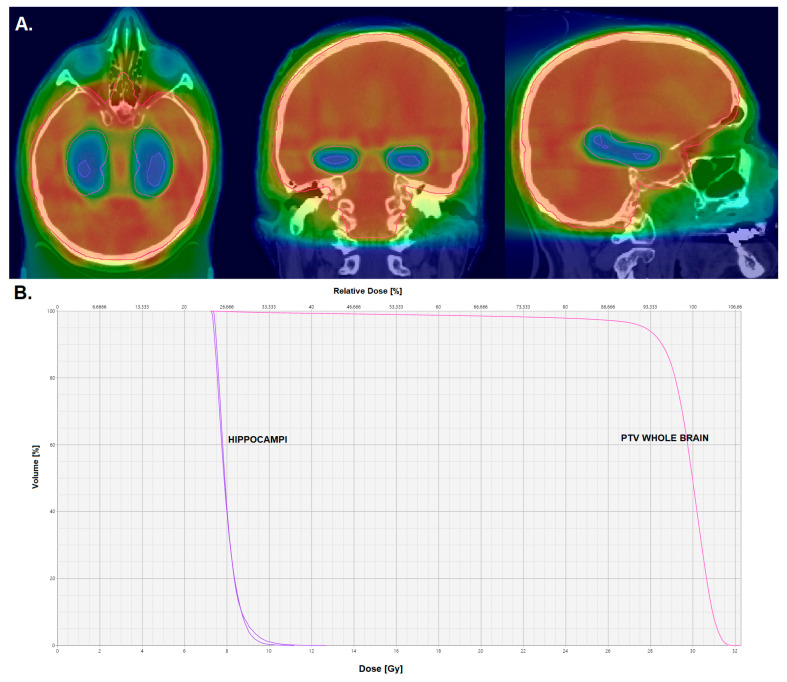
Dose distribution (**A**) and dose–volume histogram (**B**) for a plan for hippocampus-avoidance whole-brain radiation therapy.

Hippocampal dose was found to be correlated with neurocognitive function impairment, with a significant threshold at 7.3 Gy to 40% of the bilateral hippocampus applied in 2 Gy fractions [43]. The first single-arm prospective trial, developed by the RTOG 0933 consortium, proved the feasibility of simultaneous de-escalation of the dose to the hippocampus during WBRT and set hippocampus-contouring guidelines and constraints for the protection of the hippocampi [44]. The subsequent randomized phase III NRG Oncology CC001 trial proved the significant reduction in the risk of cognitive failure through hippocampal sparing (hazard ratio = 0.74 [45,46]), and other studies have confirmed the feasibility and/or the efficacy of this method in other contexts as well (Table 2). Hippocampal sparing is undertaken using either LINAC-based IMRT and VMAT techniques or helical tomotherapy and involves the definition of a 5–10 mm expansion around the hippocampus—the hippocampal avoidance region—in order to achieve maximal hippocampus protection. A typical dose distribution for a HA-WBRT plan is depicted in Figure 1.

Reducing the dose to the hippocampi has been shown to be effective in preventing cognitive decline. In accordance with the data on neurocognitive outcomes, a retrospective study investigating hippocampal volume loss before and after radiation showed threefold lower hippocampal atrophy over the course of four years following HA-WBRT as compared to conventional WBRT [47]. The latter led to an annual atrophy rate of approximately 5% in the first two years, higher than the reported mean annualized rates of 3.5–4% for patients with Alzheimer’s disease and higher than the values noticed in elderly patients experiencing worsening cognitive decline [32]. HA-WBRT also led to hippocampal atrophy with a significantly lower rate of 1.6% per year, a value which is still higher than what would be expected for the age group of the cohort [48]. Consequently, it appears that the best possible hippocampal avoidance should be achieved in order to minimize negative effects.

With a significant dose reduction, hippocampal sparing raises the risk of decreasing tumor control and could thus reduce the benefit of WBRT. However, the trials performed so far have detected a risk of 2–7% for new metastases in the hippocampus itself and of 3–11% for the hippocampal avoidance region [24,44,49]. The risk of hippocampal relapse is therefore relatively low and, taking into account the availability of salvage options, rather unproblematic. Furthermore, overall and progression-free survival rates did not differ between arms in the phase III NRG Oncology CC001 trial [45]. All in all, HA-WBRT does not appear to impair the overall oncological outcome as compared to conventional WBRT, but it does offer significant protection from neurocognitive decline.

### 3.4. Hippocampus-Avoidance Whole-Brain Radiation Therapy with Simultaneous Integrated Boost to Metastases

Conventional WBRT involves the application of a moderate dose of generally 30 Gy in 10 fractions homogeneously to the whole brain. However, this dose is not enough to treat metastases of larger size. Intracranial progressive disease has been associated with a worsening of cognition in more than one trial, with local tumor control being the most important factor for stabilizing neurocognitive function [50,51,52,53]. As a solution, dose escalation in the form of sequential SRS has been proven to increase local tumor control [24,54].

Other causes of cognitive dysfunction can include radiation-induced leukoencephalopathy and atrophy of significant cortical areas, such as the prefrontal cortex. These are more prevalent with higher total doses of radiotherapy [55,56] and could potentially be reduced by lowering the whole-brain dose.

**Figure 2:**
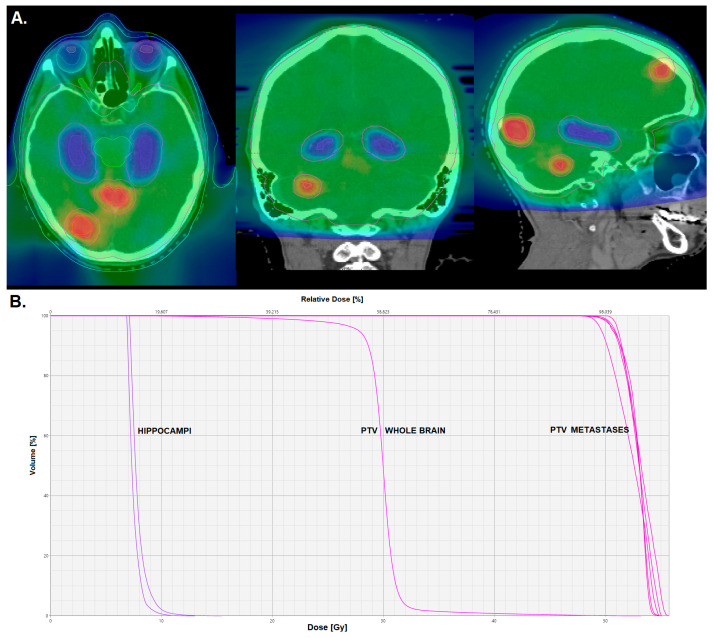
Dose distribution (**A**) and dose–volume histogram (**B**) for a plan for hippocampus-avoidance whole-brain radiation with simultaneous integrated boost to the metastases.

Aiming to address the issues of moderate local tumor control of existing metastases and whole-brain toxicity, a new method combining HA-WBRT with 30 Gy in 12 fractions and the simultaneous integrated boost (SIB) technique has been developed (Figure 2) [57]. This method is currently being explored in the prospective, randomized, multicenter phase II HIPPORAD trial (“Whole-brain irradiation with hippocampal sparing and dose escalation on metastases: neurocognitive testing and biological imaging”, NOA-14, ARO 2015-3, DRKS00004598). The trial compares HA-WBRT + SIB with WBRT + SIB without hippocampus avoidance and the results are expected in 2023.

Planning the escalation of the dose to the metastases as an SIB with 42–51 Gy in 12 fractions rather than sequential SRS allows for better sparing of the hippocampi and has the biological benefit of fractionation [54]. The lower whole-brain dose is hypothesized to reduce global cognitive decline. The dose constraints for the hippocampus are D98% lower than 9 Gy and D2% lower than 17 Gy [55].

In a pilot cohort treated according to the HIPPORAD method, the intracranial tumor control of existing metastases was shown to be significantly higher as compared to conventional WBRT, with values at one year of up to 98% [24]. However, the lower dose to the whole brain also seemed to lead to lower distant intracranial tumor control [24]. Whether the combination of hippocampus avoidance and a lower whole-brain dose can prevent cognitive decline and maintain an acceptable oncological outcome must be further investigated, which is the goal of the prospective HIPPORAD trial.

**Table 2:** Clinical trials of hippocampus-avoidance whole-brain radiation therapy for multiple brain metastases in solid tumors.

Trial	Design	Hippocampal Constraints	Patients	Neurocognitive Outcome
Gondi et al., 2014 [44]	HA-WBRT + memantine	D100% ≤ 9 Gy and Dmax ≤ 16 Gy in 10 Fx	42 (analyzable)	Hopkins Verbal Learning Test—Revised: delayed recall decline at 4 mo—7% vs. 30% (historical control), *p* < 0.001
Brown et al., 2020 [45]	HA-WBRT + memantine vs. WBRT + memantine	D100% ≤ 9 Gy and Dmax ≤ 16 Gy in 10 Fx	261 vs. 257	Cognitive failure: hazard ratio—0.76, *p* = 0.03
				Executive function at 4 mo: 23.3% vs. 40.4%, *p* = 0.01
				Learning at 6 mo: 11.5% vs. 24.7%, *p* = 0.049
				Memory at 6 mo: 16.4% vs. 33.3% *p* = 0.02
Grosu et al., 2020 [58]	HA-WBRT + SIB vs. WBRT + SIB	D98% ≤ 9 Gy and D2% ≤ 17 Gy in 12 Fx	66 vs. 66 (planned)	Results pending
Redmond et al., 2017 [59]	HA-PCI	Dmean < 8 Gy in 10 Fx	17 (analyzable)	Hopkins Verbal Learning Test—Revised: decline at 6 and 12 mo—no significant decline compared to baseline
Rodríguez de Dios et al., 2021 [60]	HA-PCI vs. PCI	D100% ≤ 9 Gy and Dmax ≤ 16 Gy in 10 Fx	75 vs. 75	Free and Cued Selective Reminding Test: delayed recall decline at 3 mo—5.8% vs. 23.5%, *p* = 0.003;
				total recall decline at 3 mo—8.7% vs. 20.6%;
				delayed recall decline at 6 mo—11.1% vs. 33.3%;
				total recall decline at 6 mo—20.3% vs. 38.9%;
				total recall decline at 24 mo—14.2% vs. 47.6%
Belderbos et al., 2021 [61]	HA-PCI vs. PCI	Dmean ≤ 8.5 Gy and D1% ≤ 10 Gy in 10 Fx	84 vs. 84	Hopkins Verbal Learning Test—Revised: total recall decline at 4 mo—28% vs. 29%, *p* = 1.000

HA-WBRT—hippocampus-avoidance whole-brain radiation therapy, HA-WBRT + SIB—hippocampus-avoidance whole-brain radiation with simultaneous integrated boost, PCI—prophylactic cranial irradiation, HA-PCI—hippocampus-avoidance prophylactic cranial irradiation, D100%—dose to 100% of the volume, Dmax—maximal dose, D98%—dose to 98% of the volume, D2%—dose to 2% of the volume, Dmean—mean dose, D1%—dose to 1% of the volume, Fx—fractions, mo—months.

## 4. Whole-Brain Radiation Therapy for Small Cell Lung Cancer

### 4.1. Prophylactic Cranial Irradiation

A special case of WBRT is prophylactic cranial irradiation (PCI) for small cell lung carcinoma (SCLC).

Approximately 50% of SCLC patients develop brain metastases during the course of their disease. The meta-analysis by Auperin et al. (1999) proved the impact of PCI in reducing the incidence of brain metastases to 33% at three years. PCI was there shown to also improve survival by 5% in patients with limited disease who had had a complete response after radiochemotherapy [62]. To date, no further randomized data proving otherwise have been published. Therefore, PCI remains the standard therapy for these patients. The usual dose is 25 Gy in 10 fractions or 30 Gy in 15 fractions [63].

For patients with extensive disease or poor response to initial treatment, performing PCI may be taken into consideration. The trial by Slotman et al. showed a reduction in the incidence of symptomatic brain metastases and an improvement in disease-free and overall survival [64]. However, the trial was criticized with respect to the lack of MRI screening, as some asymptomatic patients who nevertheless had radiologically detectable brain metastases may have been included and may have thus influenced the outcome [65]. A Japanese trial investigated PCI versus observation with MRI every three months in extensive-disease SCLC patients with no brain metastases in baseline MRI. In this trial, PCI significantly decreased the incidence of brain metastases but did not result in longer overall survival. As a consequence, PCI can be omitted for this population but may be offered to patients for whom regular 3-month MRI follow-up is not feasible.

While studies have shown no difference in cognitive function between PCI and observation [66,67], the trend of de-escalating therapy based on the fear of neurotoxic effects can also be seen here. Hippocampus-avoidance PCI (HA-PCI) is a therapeutic alternative that has shown predominantly favorable oncological outcomes and a positive impact on neurocognition in two trials [59,60]. The results of another clinical trial, however, differed from those of the previous two and showed no cognitive benefit from hippocampus avoidance [61]. One confounding variable that may explain these results is impaired baseline cognitive function, as has been seen in almost half the number of patients with SCLC [66]. Lack of cognitive reserves can explain the lack of benefit from hippocampus avoidance, as was also seen in the NRG Oncology CC001 trial [45,46]. Both studies did nevertheless confirm the safety of hippocampus avoidance, with no difference in the incidence of brain metastases as compared to conventional PCI. For a better understanding of the role of HA-PCI in SCLC, the results of the randomized NRG-CC003 trial (NCT02635009) are awaited.

### 4.2. Therapeutic Whole-Brain Radiation Therapy

For SCLC, WBRT is the first therapeutic option, even with a limited number of brain metastases. The retrospective FIRE-SCLC study investigated the option of first-line SRS and compared the outcome with that for a cohort treated with WBRT [68]. SRS did not lead to a decrease in overall survival, but the time to central nervous system progression was significantly shorter and overall survival declined with continuous increases in brain metastases. Whether WBRT can be omitted for patients with SCLC brain metastases and replaced by SRS has to be further investigated in prospective trials, such as the ongoing ENCEPHALON trial (NCT03297788).

## 5. Discussion

Based on the current literature presented above, there are two main points of criticism for WBRT: neurocognitive toxicity and the lack of benefit for overall survival. While these points have been extensively proven in patients with one to four brain metastases, there are very little to no data supporting the omission of WBRT for patients with multiple brain metastases.

To the best of our knowledge, there are no published randomized clinical trials comparing SRS and WBRT only in patients with more than four brain metastases. The advantages and, most importantly, the disadvantages of WBRT versus SRS have been derived from the available studies with one to four brain lesions. However, taking into account that multiple brain metastases may imply a different velocity, this condition should be considered and researched separately. Whether multiple SRSs, higher rates of intracranial distant progression, and frequent salvage treatments can ensure better quality of life than WBRT has to be investigated prospectively.

Furthermore, all of the trials published so far have compared SRS with conventional WBRT. There are no published data comparing the effect of SRS with those of modern WBRT techniques—HA-WBRT or HA-WBRT + SIB—on cognitive functions and oncological outcomes. Table 3 presents a selection of ongoing trials investigating SRS versus WBRT with and without hippocampus avoidance and SIB in patients with extensive intracranial metastatic disease. Their results will provide more clarity regarding the optimal treatment for this category of patients.

## 6. Conclusions

There is currently a clear trend toward replacing conventional WBRT with local stereotactic irradiation. While this has become the standard for patients with one to four metastases, the omission of WBRT for all patients with more than four metastases is still under investigation. Which patients will most probably benefit from WBRT and which from local SRS alone is still an object of current research. It is, however, important to note that tumor growth in the brain has the most important negative impact on cognition [50,53]. Proper local tumor control is, therefore, paramount to obtain the best functional results. Whether this can be achieved with WBRT or local stereotactic treatment has to be carefully assessed individually for each patient. If WBRT is chosen, modern planning with hippocampal avoidance and dose escalation to the metastases should be favored whenever possible. Prospective trials comparing multiple SRSs with the modified HA-WBRT +/− SIB techniques are on the way and should help further define the optimal therapy for this group of patients.

## Figures and Tables

**Table 3:** Ongoing trials comparing radiosurgery with conventional and modern whole-brain radiation therapy techniques (selection).

Trial	Number of Brain Metastases	Study Arms
WHOBI-STER (NCT04891471)	≥5	SRS vs. WBRT
Sunnybrook (NCT03775330)	5–30	SRS vs. SRS + WBRT
MDACC (NCT01592968)	≥5	SRS vs. WBRT
HIPPORAD-RS (DRKS00025906)	4–10	SRS vs. HA-WBRT +/− SIB
HipSter (NCT04277403)	4–15	SRS vs. HA-WBRT + SIB
CCTG CE.7 (NCT03550391)	≥5	SRS vs. HA-WBRT
NRG Oncology (NCT04804644)	≤10 (SCLC)	SRS vs. HA-WBRT

SRS—stereotactic radiosurgery, WBRT—whole-brain radiation therapy, HA-WBRT—hippocampus-avoidance whole-brain radiation therapy, SIB—simultaneous integrated boost, SCLC—small cell lung cancer.

## Data Availability

Not applicable.
